# Identification and validation of aging-related genes in neuropathic pain using bioinformatics

**DOI:** 10.3389/fgene.2024.1430275

**Published:** 2024-07-24

**Authors:** Hui Gao, Guoqi Dong, Yong Yao, Huayuan Yang

**Affiliations:** School of Acupuncture-Moxibustion and Tuina, Shanghai University of Traditional Chinese Medicine, Shanghai, China

**Keywords:** neuropathic pain, machine learning, aging, diagnostic biomarkers, bioinformatics

## Abstract

**Background:**

Neuropathic pain (NP) is a debilitating and refractory chronic pain with a higher prevalence especially in elderly patients. Cell senescence considered a key pathogenic factor in NP. The objective of this research is to discover genes associated with aging in peripheral blood of individuals with NP using bioinformatics techniques.

**Methods:**

Two cohorts (GSE124272 and GSE150408) containing peripheral blood samples of NP were downloaded from the GEO database. By merging the two cohorts, differentially expressed aging-related genes (DE-ARGs) were obtained by intersection with aging-related genes. The potential biological mechanisms of DE-ARGs were further analyzed through GO and KEGG. Three machine learning methods, namely, LASSO, SVM-RFE, and Random Forest, were utilized to identify diagnostic biomarkers. A Nomogram model was developed to assess their diagnostic accuracy. The validation of biomarker expression and diagnostic effectiveness was conducted in three distinct pain cohorts. The CIBERSORT algorithm was employed to evaluate the immune cell composition in the peripheral blood of patients with NP and investigate its association with the expression of diagnostic biomarkers.

**Results:**

This study identified a total of 24 DE-ARGs, mainly enriched in “Chemokine signaling pathway,” “Inflammatory mediator regulation of TRP channels,” “HIF-1 signaling pathway” and “FOXO signaling pathway”. Three machine learning algorithms identified a total of four diagnostic biomarkers (CEBPA, CEACAM1, BTG3 and IL-1R1) with good diagnostic performance and the similar expression difference trend in different types of pain cohorts. The expression levels of CEACAM1 and IL-1R1 exhibit a positive correlation with the percentage of neutrophils.

**Conclusion:**

Using machine learning techniques, our research identified four diagnostic biomarkers related to aging in peripheral blood, providing innovative approaches for the diagnosis and treatment of NP.

## 1 Introduction

In the extensive cross-sectional study of middle-aged adults in the UK Biobank, neuropathic pain (NP), a chronic pain condition resulting from somatosensory nervous system damage or disease, was found to have a prevalence of 9.2%, comprising 18.1% of individuals with chronic pain (Baskozos et al., 2023). It is characterized by symptoms such as paresthesia, dysesthesias, allodynia, hyperalgesia, and spontaneous unprovoked pain, significantly impacting quality of life (Woolf and Mannion, 1999). NP is predominantly diagnosed and assessed through questionnaire reports in clinical practice. Nevertheless, these assessments have inherent limitations, such as the potential failure to detect up to 20% of NP cases depending on the specific circumstances, as well as the brevity or absence of clinical examinations (Attal et al., 2018; Attal et al., 2023). Besides, the etiology of NP is multifaceted, and the optimal treatment approach remains elusive (Attal and Bouhassira, 2021). Hence, the discovery of biomarkers exhibiting elevated sensitivity and efficacy may aid in the prompt detection of NP and enhance the exploration of NP pathogenesis for personalized therapeutic interventions.

Cell senescence is recognized as a contributing factor to NP development. Age-related changes increase vulnerability to pain, affecting pain tolerance and the ability to recover from injuries (Mullins et al., 2022). The incidence of NP in the elderly demographic has been documented to be significantly elevated, ranging from 32% to 40%, and is likely to be underestimated (Pickering et al., 2016; Rapo-Pylkkö et al., 2016; Stompór et al., 2019). Previous study has indicated that male mice showed reduced telomere length and p53-mediated cellular senescence in the spinal cord, resulting in prolonged pain and a link to decreased lifespan (Muralidharan et al., 2022). Besides, analgesics frequently employed in clinical practice exhibit restricted efficacy in elderly individuals experiencing NP, primarily due to adverse effects (Fu and Perloff, 2022). However, the specific mechanism by which cell senescence is involved in NP remains unclear.

The rapid progression of bioinformatics technology has equipped researchers with the capability to identify alterations in gene expression data among various types of pain. Previous study have explored the potential impact of aging on NP through the intersection of NP and aging-related differentially expressed genes in dorsal root ganglia (Ye et al., 2022). However, sampling of diseased nerve tissue causes great damage to the patient and is not conducive to the early diagnosis of NP. Certain studies have demonstrated the existence of reliable and consistent biomarkers in the peripheral blood of NP patients (Thakkar and Acevedo, 2023). Therefore, this study applied peripheral blood samples of NP obtained from GEO and used three machine learning algorithms to obtain NP diagnostic biomarkers related to aging genes, providing new ideas for the early diagnosis of NP.

## 2 Methods and materials

### 2.1 Dataset acquisition and preparation

The Whole-blood RNA-seq transcriptome data from various pain types (GSE124272, GSE150408, GSE151371, GSE95849, GSE93272) in peripheral blood samples were retrieved from the Gene Expression Omnibus (GEO) database (http://www.ncbi.nlm.nih.gov/geo/) (Table 1). The cohorts GSE124272 comprises 8 NP patients and 8 healthy individuals, while GSE150408 comprises 17 NP patients and 17 healthy individuals. Additionally, it should be noted that the treated patients are excluded from the GSE150408 dataset. GSE151371 includes 38 patients with spinal cord injury and 10 healthy individuals, GSE95849 includes 6 patients with diabetic peripheral neuropathy and 6 healthy individuals, GSE93272 includes 232 patients with rheumatoid arthritis and 43 healthy individuals. Utilizing R software (version 4.3.0), all analyses and visualizations were conducted. The two series matrix files (GSE124272 and GSE150408) underwent annotation with the microarray platform, resulting in gene expression matrix files. Subsequently, the two gene expression matrix files were merged into a single file, and batch normalization of the expression data from the two distinct cohorts was performed using the “sva” R package. This process yielded a normalized gene expression matrix file, which contained data from two cohorts, facilitating the analysis of differentially expressed genes (DEGs). Meanwhile, GSE151371, GSE95849 and GSE93272 were used as the validation cohorts. The Perl software (version 5.30) was employed to annotate microarray probe names with gene symbols, utilizing the average value when multiple probes mapped to a single gene symbol. All gene expression values have been normalized and transformed by Log2 (x+1).

**TABLE 1:** The details of gene expression datasets.

Dataset	Platform	Tissue	Type of pain	Species	Health	Patients
GSE124272	GPL21185	Peripheral blood	Neuropathic pain	Homo sapien	8	8
GSE150408	GPL21185	Peripheral blood	Neuropathic pain	Homo sapien	17	17
GSE151371	GPL20301	Peripheral blood	Neuropathic pain	Homo sapien	10	38
GSE95849	GPL22448	Peripheral blood	Neuropathic pain	Homo sapien	6	6
GSE93272	GPL570	Peripheral blood	Inflammatory Pain	Homo sapien	43	232

### 2.2 Differentially expressed age-related genes (DE-ARGs)

Differential expression analysis was conducted using the “limma” package in R software with the criteria of *p*-value <0.05 and abs (logFC) > 0.35. Visualization of volcano plots and heatmaps for differentially expressed genes (DEGs) was achieved with the “ggplot2” and “heatmap” packages. Aging-related genes sourced from the Human Ageing Genomic Resources (HAGR, https://genomics.senescence.info/) (Supplementary Table S1) were integrated, resulting in 466 genes for analysis. The overlapping genes between DEGs and aging-related genes were identified as DE-ARGs and depicted in a Venn diagram generated by the “VennDiagram” package.

### 2.3 GO and KEGG pathway enrichment analysis

Enrichment analysis of Gene Ontology (GO) and Kyoto Encyclopedia of Genes and Genomes (KEGG) was carried out using the “clusterProfiler” package (version 4.8.1) with the org. Hs.eg.db background (version 3.17.0). GO analysis encompassed biological processes (BP), cellular components (CC), and molecular function (MF). Terms from GO and KEGG with an adjusted *p*-value <0.05 were deemed statistically significant.

### 2.4 Best gene biomarkers for the diagnosis of NP

Three machine learning algorithms, namely, Random Forest, least absolute shrinkage and selection operator (LASSO), and support vector machine recursive feature elimination (SVM-REF), were utilized to explore significant diagnostic biomarkers for NP. Random forest is a versatile predictive algorithm that leverages ensemble learning to integrate multiple trees, yielding accurate predictions despite variable conditions. It was implemented using the “randomForest” package with a parameter setting of nTree = 500 and the top 10 genes were identified based on mean decrease Gini (MDG). The LASSO algorithm was executed through the “glmnet” package. It reduces dimensions by including independent variables with non-zero coefficients, preventing overfitting in high-dimensional data and aiding variable selection, allowing identification of characteristic genes. SVM-RFE is a prevalent supervised machine-learning protocol that identifies important variables by discarding SVM-generated eigenvectors, enhancing discriminative power of biomarkers for classification and regression tasks. It was conducted using the “e1071” package, and mean misjudgment rates were assessed through 10-fold cross-validation. The final set of NP-related diagnostic biomarkers was derived by intersecting the results from the three machine learning algorithms.

### 2.5 Establishment and evaluation of nomogram

ROC curves were generated using the “pROC” package (version 1.18.0) to assess the diagnostic value of biomarkers for NP, with AUC and 95% confidence intervals calculated. The nomogram, created with the “rms” package, displayed gene expression scores on the plot for NP evaluation. Subsequently, the ROC curve based on the nomogram model was plotted. AUC >0.7 indicates moderate diagnostic value, with AUC >0.9 indicating high diagnostic value. Additionally, a calibration curve demonstrated the discriminatory efficacy of the nomogram for NP, and the decision curve analysis (DCA) assessed the net clinical benefit of the models.

### 2.6 Immune infiltration and immune-related factors

**FIGURE 1:**
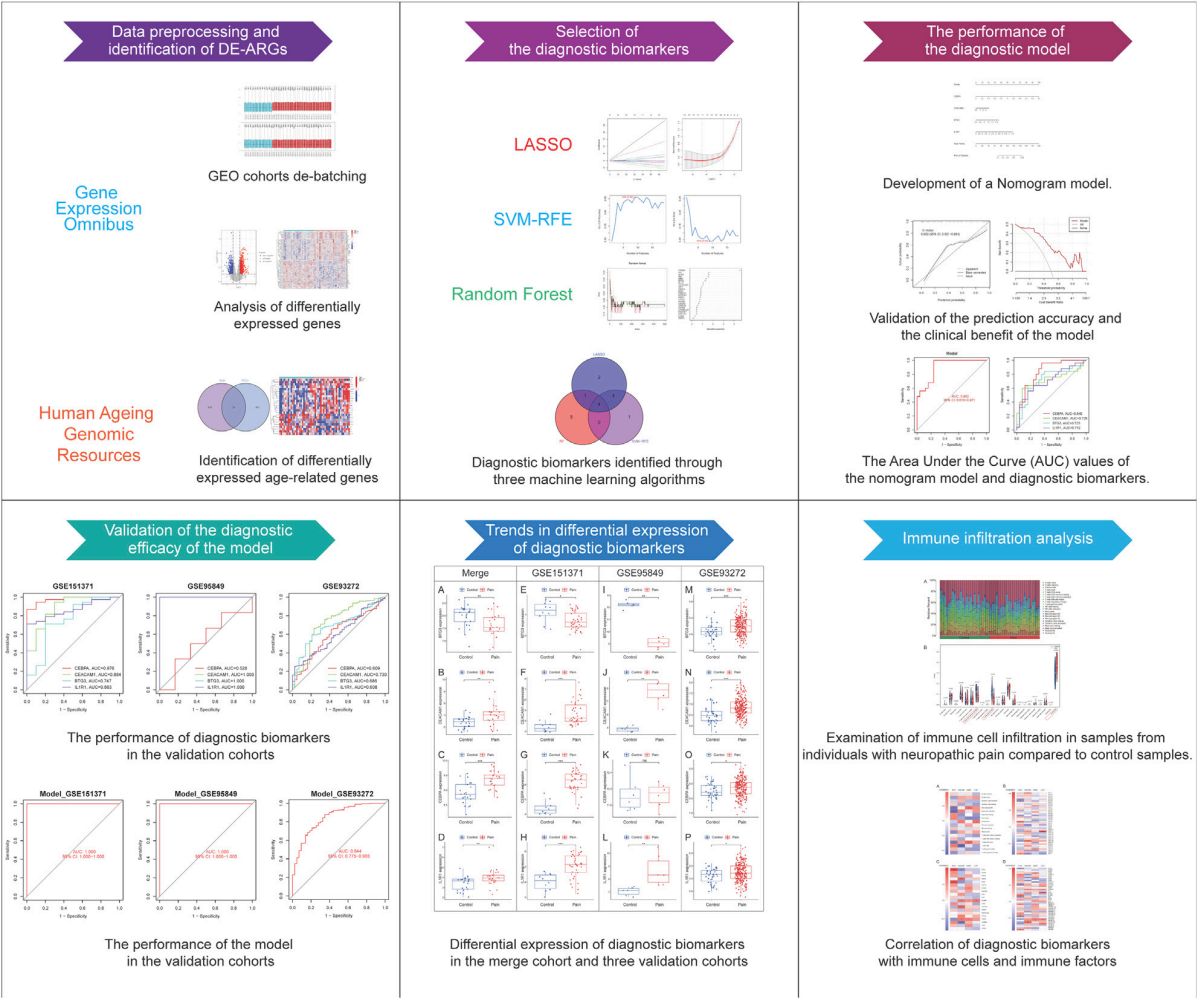
Work flowchart.

The CIBERSORT algorithm was utilized to measure immune cell infiltration in NP samples. Following the exclusion of samples with non-significant results (*p*-value >0.05), the Wilcoxon test was employed to assess the variations in 22 immune cell populations between individuals with NP patients and healthy controls. Additionally, immune factors data were sourced from the TISIDB database (http://cis.hku.hk/TISIDB), (Ru et al., 2019) as detailed in Supplementary Table S2. Spearman correlation analysis was conducted to explore the associations between diagnostic biomarkers and immune entities. The bioinformatics procedures of this investigation are illustrated in Figure 1.

## 3 Results

### 3.1 Data preprocessing and identification of DE-ARGs

**FIGURE 2:**
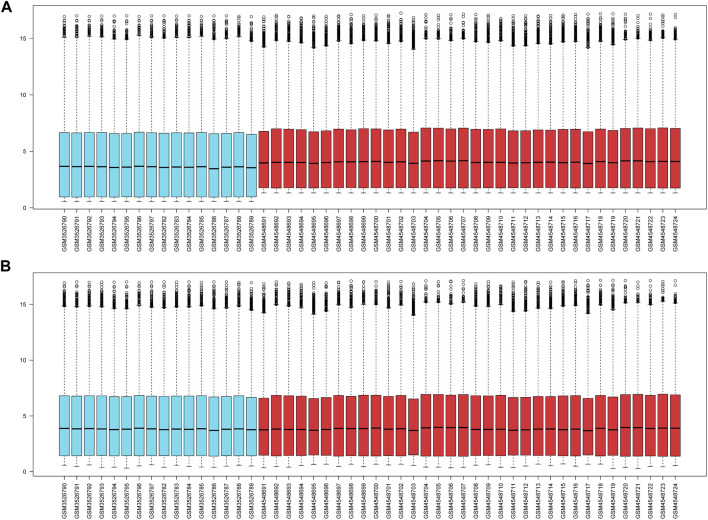
**(A)** The gene expression levels of two cohorts prior to the de-batching process. **(B)** The gene expression levels of the integrated cohort following the process of de-batching.

**FIGURE 3:**
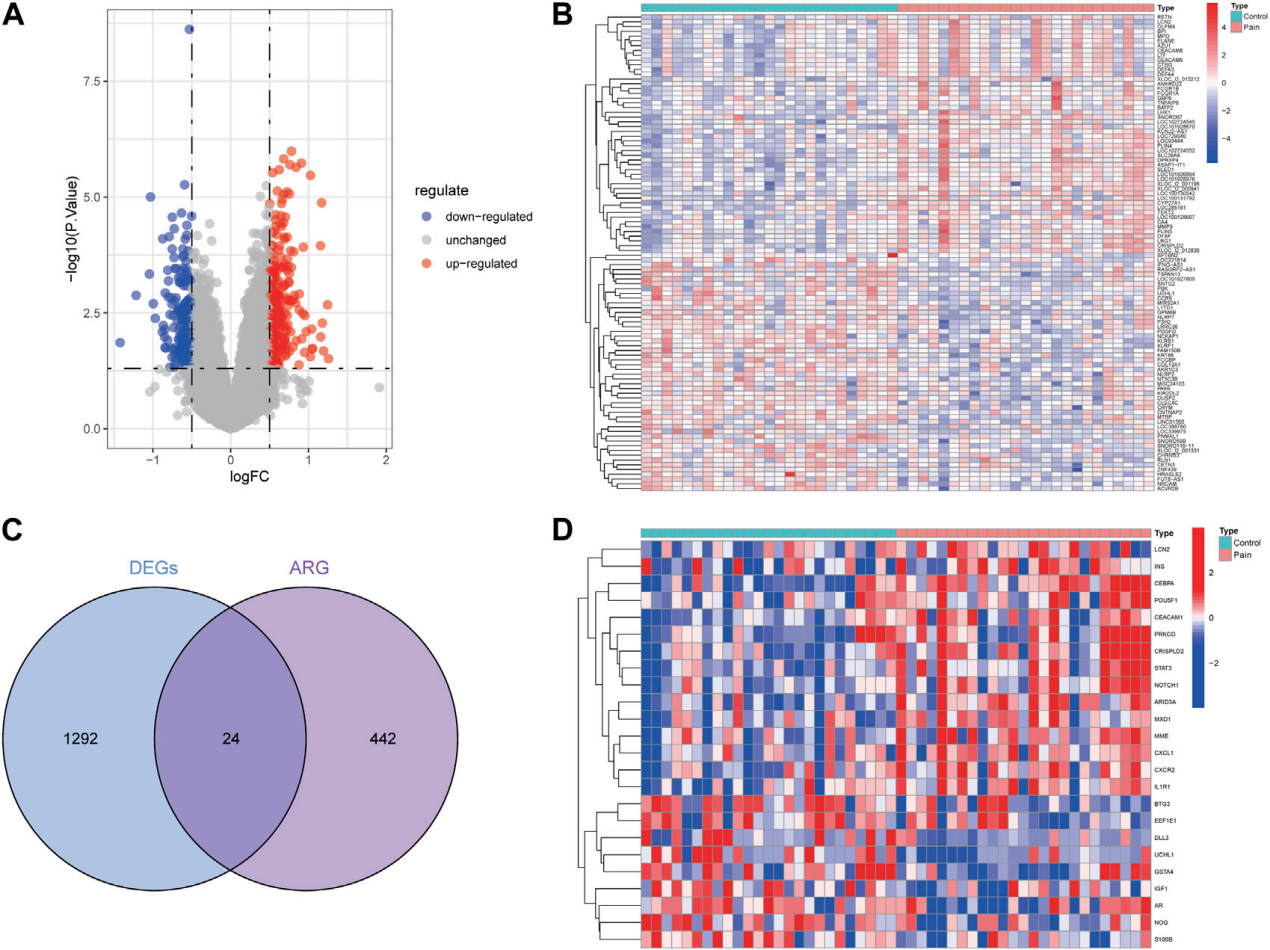
Identification of aging-related genes related to NP. The volcano plot **(A)** and heatmap **(B)** for DEGs related to NP. **(C)** Intersection of aging-related genes and DEGs. **(D)** Heat map of 24 DE-ARGs.

Two datasets (GSE124272 and GSE150408) based on the same platform (GPL21185) were merged into one dataset after removing the batch effect (Figure 2). According to the differential expression analysis, 1,316 DEGs were screened from the integrated dataset, including 718 upregulated and 598 downregulated genes (Figures 3A, B). We identified 24 DE-ARGs (Figure 3C), consisting of 15 upregulated and 9 downregulated genes (Figure 3D), by taking the intersection of aging-related genes and DEGs.

### 3.2 GO and KEGG enrichment analysis

**FIGURE 4:**
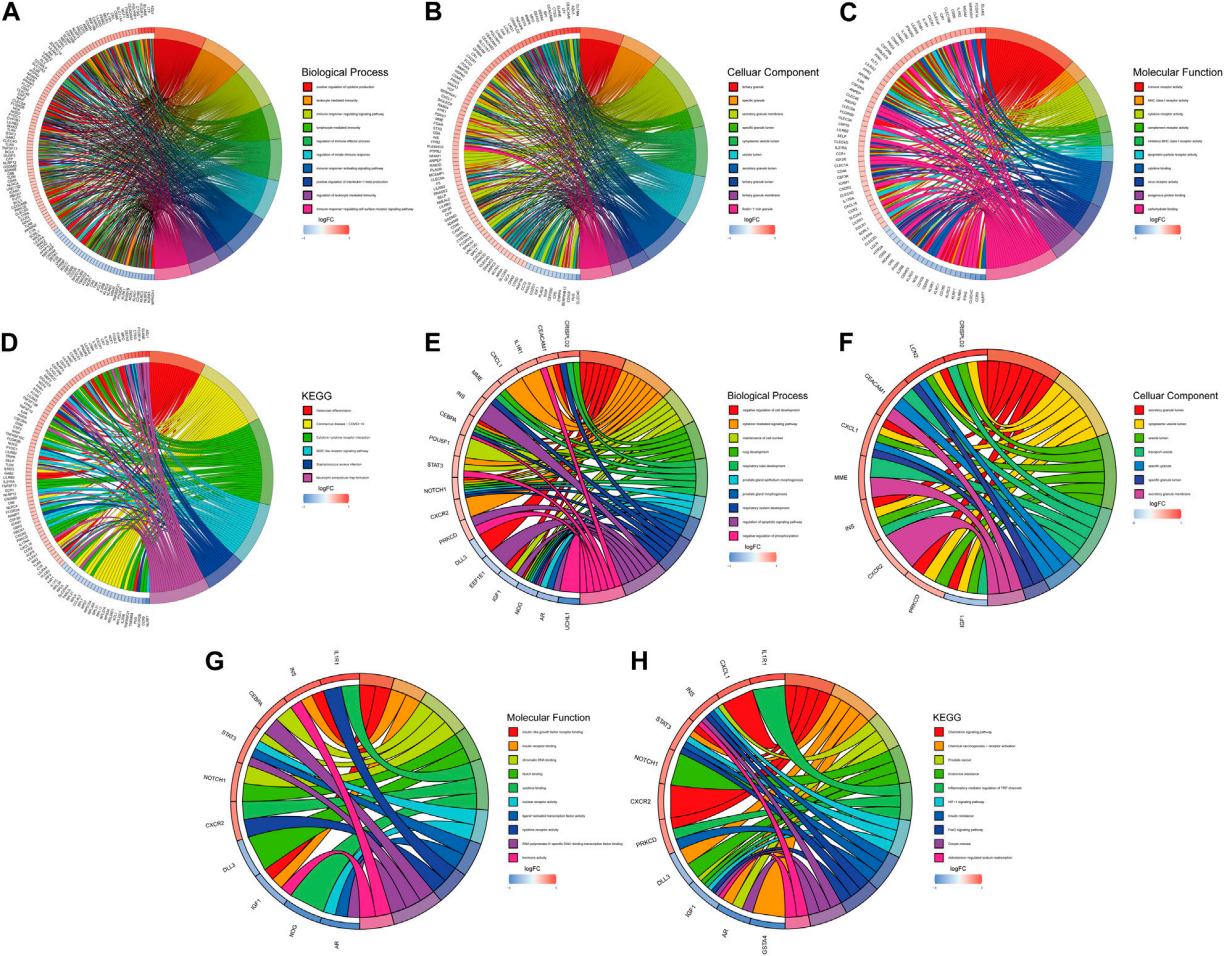
GO and KEGG analysis. Top ten biological processes **(A)**, cellular component **(B)**, molecular function **(C)** and KEGG **(D)** pathway in DEGs. Top ten biological processes **(E)**, cellular component **(F)**, molecular function **(G)** and KEGG **(H)** pathway in DE-ARGs.

We first conduct functional enrichment analysis based on DEGs. In GO analysis, the richest biological process participated “positive regulation of cytokine production,” “leukocyte mediated immunity,” and “immune response-regulating signaling pathway” (Figure 4A). In the cellular components category, DEGs participated in structures including “tertiary granule,” “specific granule,” and “secretory granule membrane” (Figure 4B). “immune receptor activity,” “MHC class I receptor activity,” and “cytokine receptor activity” were enriched for DEGs in the molecular functions category. (Figure 4C). KEGG pathway analysis revealed significant enrichment of DEGs in pathways such as “osteoclast differentiation,” “cytokine-cytokine receptor interaction,” “HIF-1 signaling pathway,” and “NOD-like receptor signaling pathway” (Figure 4D). Functional enrichment analysis was also carried out on DE-ARGs to investigate their potential functions. In the biological process category, DE-ARGs showed significant associations with “negative regulation of cell development,” “cytokine-mediated signaling pathway,” and “maintenance of cell number” (Figure 4E). In the cellular components category, DE-ARGs participated in structures including “secretory granule lumen,” “cytoplasmic vesicle lumen,” and “vesicle lumen” (Figure 4F). For molecular functions, the main roles were in “insulin-like growth factor receptor binding,” “insulin receptor binding,” and “chromatin DNA binding” (Figure 4G). KEGG pathway analysis revealed significant enrichment of DE-ARGs in pathways such as “Chemokine signaling pathway,” “Inflammatory mediator regulation of TRP channels,” “HIF-1 signaling pathway,” and “FOXO signaling pathway” (Figure 4H).

### 3.3 Selection of the diagnostic biomarkers via the machine learning algorithms

**FIGURE 5:**
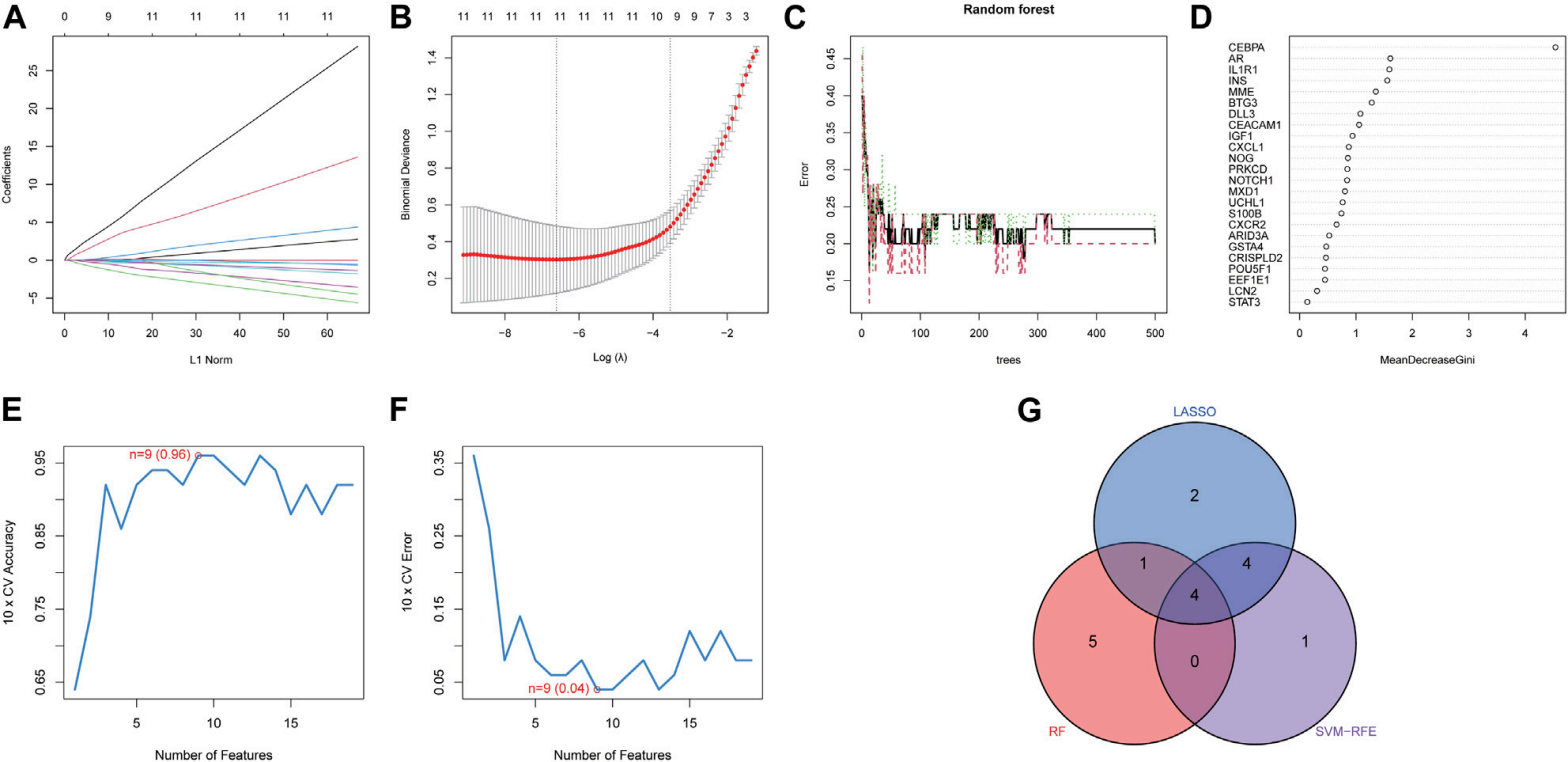
Identification of diagnostic biomarkers. The minimum **(A)** and lambda values **(B)** of diagnostic biomarkers were identified by LASSO. **(C)** Potential diagnostic biomarkers selection via Random Forest. **(D)** MeanDecreaseGini showed the rank of genes in accordance with their relative importance. **(E)** Accuracy and **(F)** error of 10-fold cross-validation in SVM-RFE algorithms, respectively. **(G)** Diagnostic biomarkers identified through the intersection of three machine algorithms.

Three distinct machine-learning algorithms were employed to identify potential diagnostic biomarkers. In the LASSO logistic regression, the penalty parameter was optimized through 10-fold cross-validation, leading to the selection of eleven DE-ARGs (Figures 5A, B). Concurrently, the Random Forest algorithm identified ten genes characterized by the highest MeanReducedGini values (Figures 5C, D). Analysis via SVM-RFE demonstrated that the leading nine DE-ARGs exhibited the lowest error rate (0.04) and the highest accuracy (0.96) in the identification of diagnostic biomarkers (Figures 5E, F). Ultimately, overlapping gene analysis from LASSO, SVM-RFE, and Random Forest facilitated the identification of four NP-related diagnostic biomarkers (CEBPA, CEACAM1, BTG3, IL1R1) for further investigation (Figure 5G).

### 3.4 The diagnostic efficacy of the biomarkers

**FIGURE 6:**
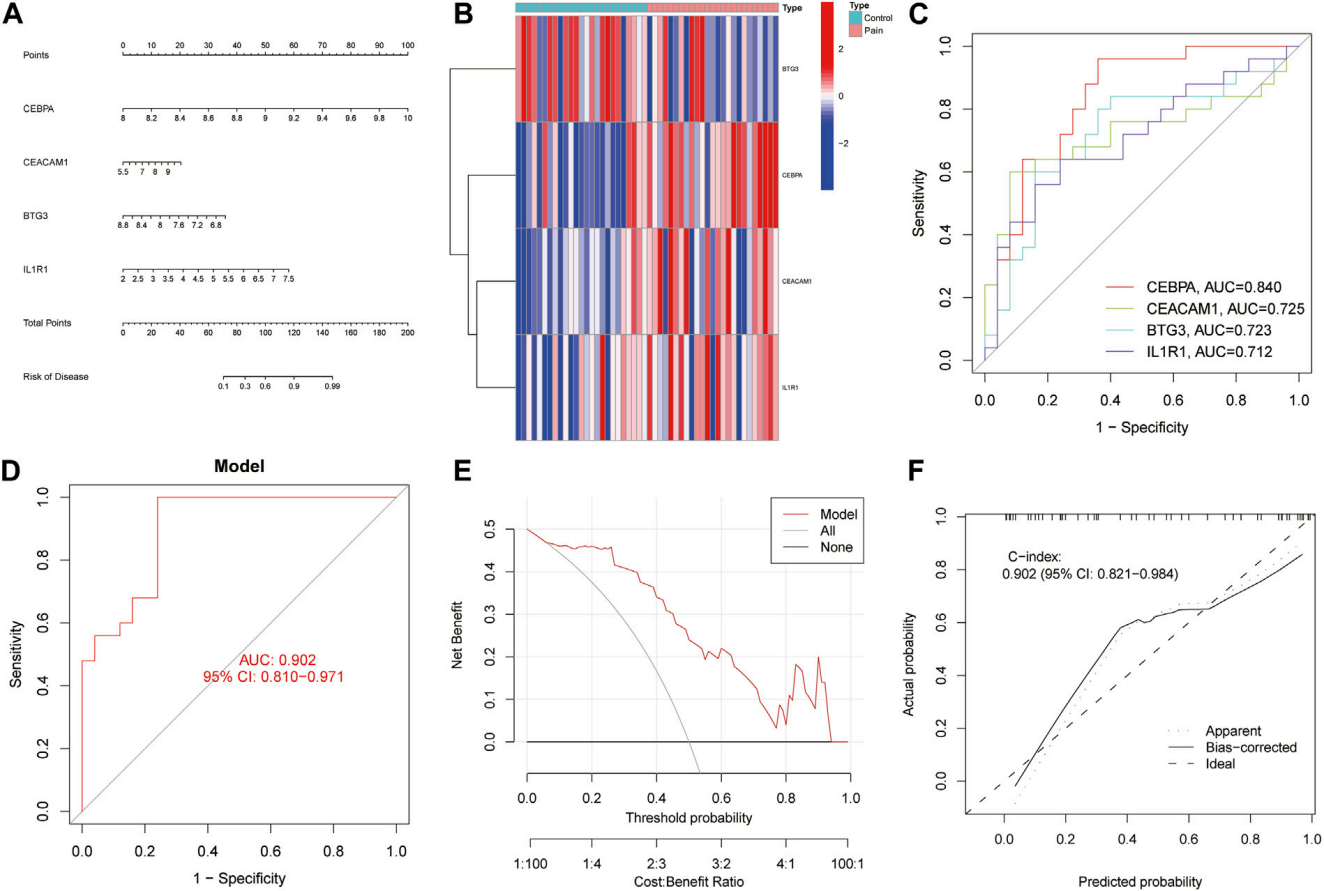
Development of the nomogram model and evaluation of its efficacy. **(A)** The nomogram depicting diagnostic biomarkers for predicting the risk of developing NP. **(B)** Heatmaps related to diagnostic biomarkers. **(C)** The ROC curve for each biomarker. **(D)** The ROC curve for the nomogram model. **(E)** DCA results to evaluate the clinical value of the nomogram model in NP. **(F)** The calibration curve of nomogram model prediction in NP.

A nomogram incorporating diagnostic biomarkers elucidates the diagnostic significance of four biomarkers for NP (Figure 6A), while a heatmap displays their differential expression between NP and control samples (Figure 6B). ROC curve analysis reveals the AUC values for the biomarkers: 0.840 for CEBPA, 0.725 for CEACAM1, 0.723 for BTG3, and 0.712 for IL1R1 (Figure 6C). Additionally, the nomogram demonstrates a high diagnostic potential for NP with an AUC of 0.902 (95% CI: 0.810–0.971) (Figure 6D). Decision Curve Analysis (DCA) indicates that the curve surpass the two benefit thresholds, highlighting its substantial efficacy (Figure 6E). Furthermore, in the calibration curve analysis, the performance of the column line plot approximates that of the ideal model, as predicted by the independent nomogram, underscoring reliable predictive value of the model (Figure 6F).

### 3.5 Validation of the diagnostic efficacy of the nomogram

**FIGURE 7:**
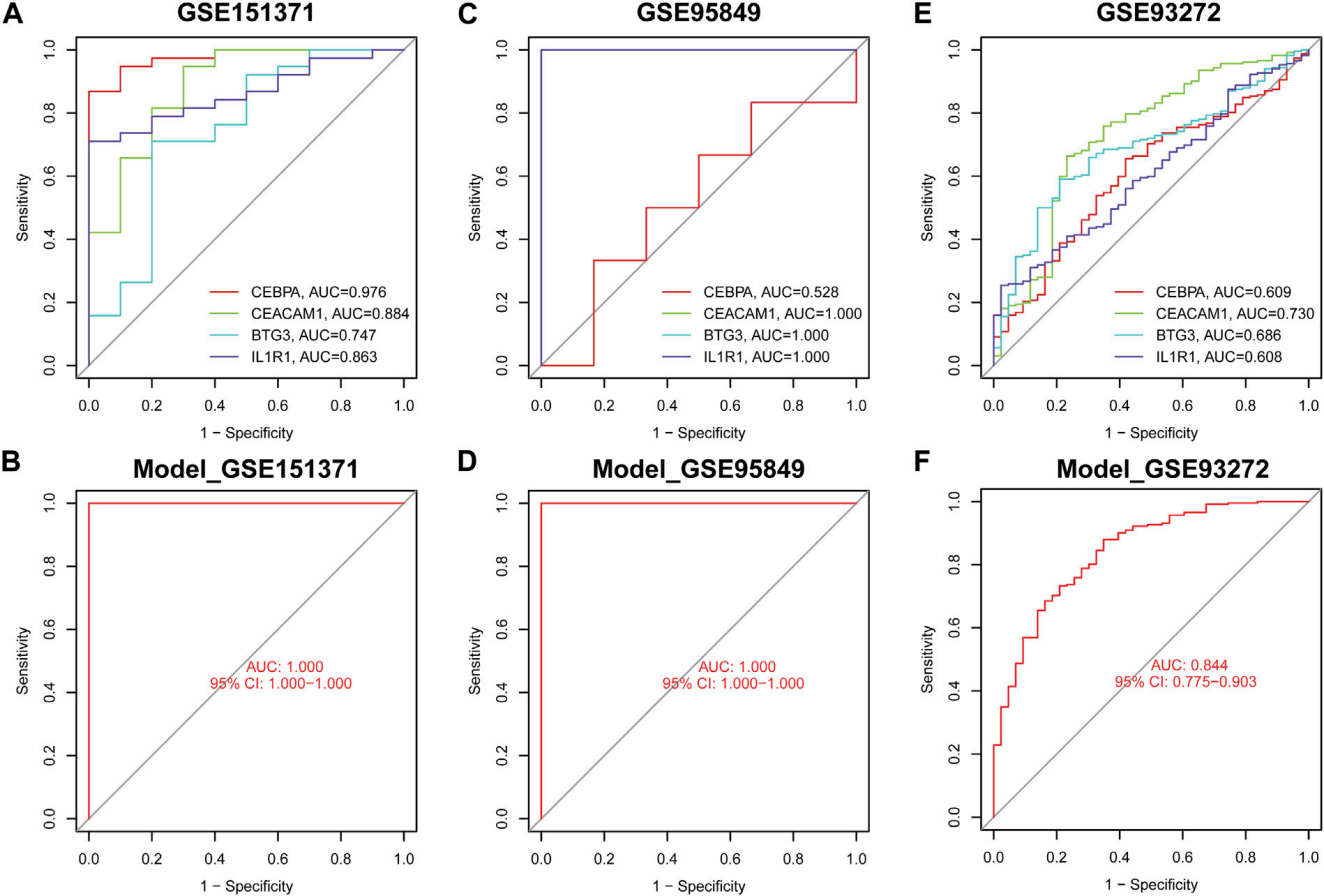
The ROC curve demonstrates the predictive accuracy of the nomogram model in validation cohorts. The ROC curves of each diagnostic biomarker in GSE175371 **(A)**, GSE95849 **(C)** and GSE93272 **(E)**. The ROC curves of the nomogram in GSE175371 **(B)**, GSE95849 **(D)** and GSE93272 **(F)**.

The diagnostic efficacy of four genes across different pain types was assessed using peripheral blood samples from cohorts with spinal cord injury (GSE151371), diabetic peripheral neuropathy (GSE95849) and rheumatoid arthritis (GSE93272). In the spinal cord injury cohort, the AUC values for CEBPA, CEACAM1, BTG3, and IL1R1 were 0.976, 0.844, 0.747, and 0.863, respectively (Figure 7A), with the diagnostic model demonstrating optimal efficacy (AUC = 1.000) (Figure 7B). Similarly, in the diabetic peripheral neuropathy cohort, AUC values for the genes CEBPA, CEACAM1, BTG3, and IL1R1 reached 0.528, 1.000, 1.000, and 1.000, respectively (Figure 7C), and the diagnostic model exhibited strong performance (AUC = 1.000) (Figure 7D). Besides, in the rheumatoid arthritis, the AUC values for CEBPA, CEACAM1, BTG3, and IL1R1 were 0.609, 0.730, 0.686, and 0.608, respectively (Figure 7E) and diagnostic model achieved a high AUC of 0.844 on this cohort (Figure 7F).

### 3.6 Differential expression trends of diagnostic biomarkers in validation cohorts

**FIGURE 8:**
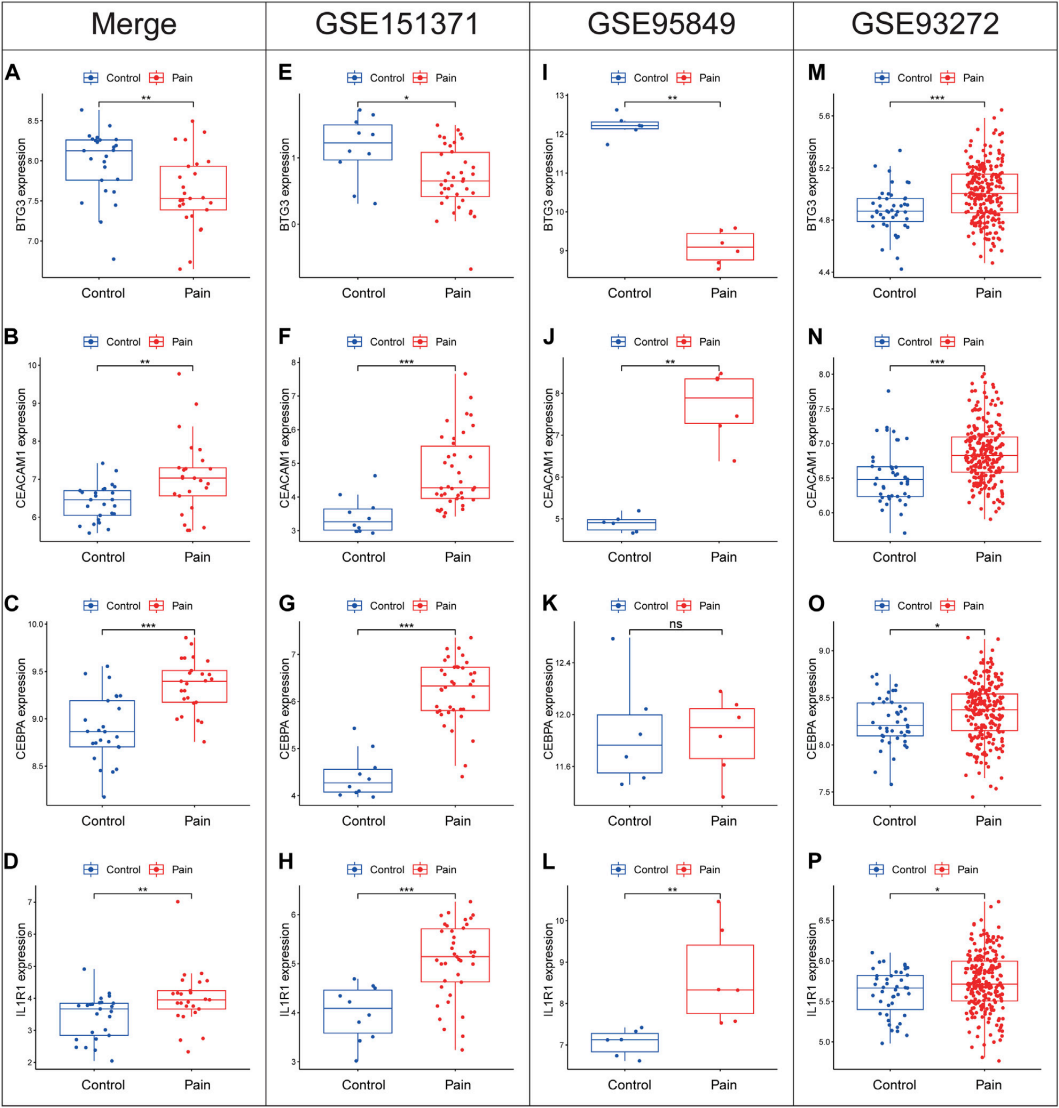
Expression levels of BTG3, CEACAM1, CEBPA and IL1R1 in the merge cohort **(A–D)**, GSE151371 **(E–H)**, GSE95849 **(I–L)** and GSE93272 **(M–P)**. *p < 0.05, **p < 0.01, ***p < 0.001.

Expression levels of diagnostic biomarkers were analyzed in validation cohorts using peripheral blood samples from GSE151371, GSE95849 and GSE93272. BTG3 exhibited downregulation across the combined cohort (Figure 8A), as well as individually in GSE151371 (Figure 8E) and GSE95849 (Figure 8I). However, BTG3 shows the opposite expression trend in GSE93272 (Figure 8M). Conversely, CEACAM1, CEBPA, and IL1R1 showed upregulation in the merged cohort (Figures 8B–D) and consistently in GSE151371 (Figures 8F–H), GSE95849 (Figures 8J–L) and GSE93272 (Figures 8N–P). Notably, CEBPA did not display significant differential expression in GSE95849. These findings indicate a consistent trend of differential expression for the four diagnostic biomarkers across all examined cohorts.

### 3.7 Analysis of immune infiltration

**FIGURE 9:**
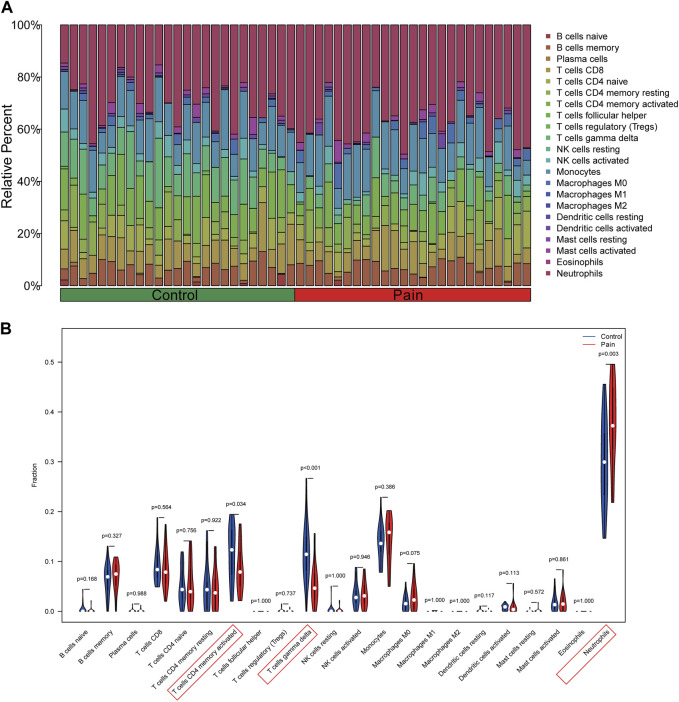
Analysis of immune infiltration in peripheral blood samples of NP patients. **(A)** The barplot illustrating the comparative distribution of various immune cell types in samples from individuals with NP patients and healthy individuals. **(B)** The violin plot comparing multiple immune cells between NP and healthy samples.

**FIGURE 10:**
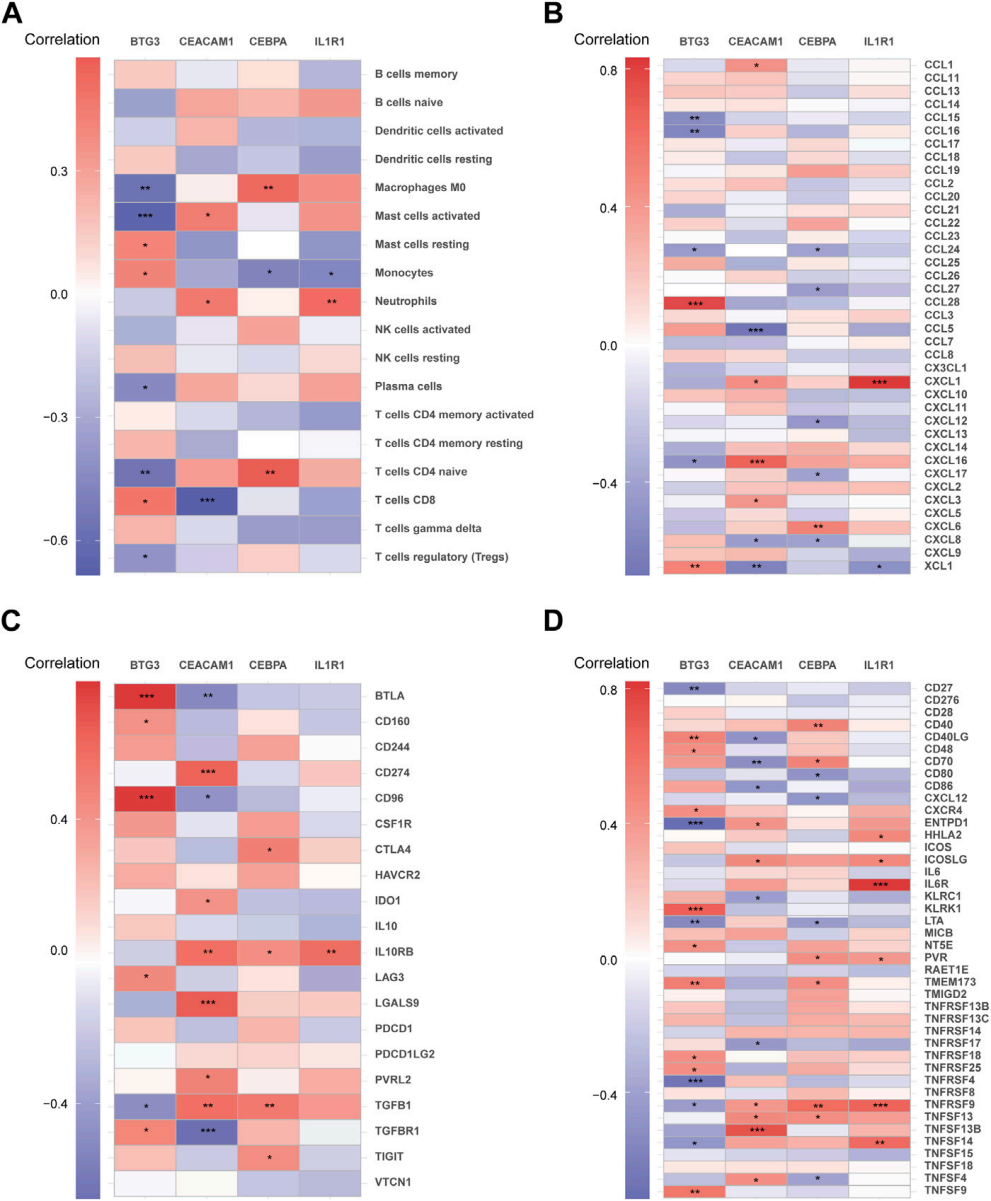
The correlation matrix depicting the relationships between diagnostic biomarkers and immune cells **(A)**, chemokines **(B)**, immunoinhibitors **(C)**, and immunostimulators **(D)**. *p < 0.05, **p < 0.01, ***p < 0.001.

Using the CIBERSORT algorithm, the relative proportions of diverse immune cells in samples from individuals with NP and healthy controls were assessed (Figure 9A). The violin plot (Figure 9B) illustrates a notable increase in neutrophil proportions in NP samples, while T cells CD4 memory activated and T cells gamma delta exhibited lower proportions. Spearman correlation analysis (Figure 10A) revealed a positive correlation between CEACAM1, IL-1R1, and neutrophils. Additionally, heatmaps demonstrated significant correlations between diagnostic biomarkers and various immune factors, including chemokines (Figure 10B), immunoinhibitors (Figure 10C), and immunostimulators (Figure 10D).

## 4 Discussion

The prevailing opinion among experts is that tailoring therapeutic interventions to individual patients offers the greatest potential for improving outcomes in the management of NP (Baron et al., 2023; Edwards et al., 2023). Patient stratification may involve the consideration of multiple biomarkers, including genetics, electrophysiology, skin punch biopsy, and imaging (Davis et al., 2020). Numerous research studies are increasingly investigating the association between cellular senescence and NP. Prior research has indicated a significant decrease in proliferating cell nuclear antigen (PCNA) mRNA expression levels within the subgranular and dentate gyrus zone of the hippocampus in chronic constriction injury (CCI) rats (Tyrtyshnaia et al., 2019), as well as in aged animals (Tyrtyshnaia et al., 2017). Besides, significant increases in the main marker of senescence β-galactosidase (β-gal) were observed in microglia in the hippocampus and spinal cord dorsal horn of spared nerve injury (SNI) mice (Borgonetti and Galeotti, 2022; Du et al., 2023). The identification of cell senescence markers in peripheral blood can aid in the detection of biomarkers and assessment of drug efficacy for various diseases such as Alzheimer’s disease (Salech et al., 2022), mild cognitive impairment (Ma et al., 2023), and diabetes (Al Dubayee et al., 2021). In fact, the accessibility of blood samples from patients has rendered peripheral blood a dependable specimen for identifying markers of cell senescence. In this study, we identified 24 DE-ARGs in peripheral blood samples from NP patients. Four diagnostic biomarkers were determined using three machine learning algorithms, and validation was performed using two external cohorts.

CCAAT enhancer binding protein alpha (CEBPA) governs lineage-specific gene expression and serves as a pivotal regulator for the terminal differentiation of various cell types. Dynamical analysis of Boolean networks demonstrated that a significant molecular mechanism contributing to the aging of hematopoietic stem cells (HSC) is the loss of CEBPA activation by Gata2 (Hérault et al., 2023). CEBPA exhibits a specific binding affinity to the CXCR3 promoter, thereby facilitating the upregulation of CXCR3 mRNA expression in the spinal cord of mice exhibiting NP. Conversely, the intrathecal administration of CEBPA siRNA results in a reduction in CXCR3 mRNA expression, consequently alleviating pain hypersensitivity induced by spinal nerve ligation (Jiang et al., 2017). Interferon-γ treatment can promote the increase of CEBPA mRNA expression in human umbilical cord mesenchymal stem cells and relieve pain in diabetic peripheral neuropathy mice (Yang et al., 2023). In addition to NP, CEBPA binding to the NGF gene promoter in the dorsal root ganglion of rats is markedly elevated, leading to an increase in NGF mRNA expression. Intrathecal injection of CEBPA siRNA can significantly reduce the expression of NGF mRNA and alleviate pain triggered by complete Freund’s adjuvant (CFA) (Yuan et al., 2020). Although there is no evidence supporting the use of CEBPA as a peripheral blood diagnostic biomarker for NP, a notable decrease in the methylation level of CpG C-289, a recognized CEBPA binding site, has been observed in the peripheral blood of patients with chronic widespread pain, indicating that CEBPA has the potential to become a peripheral blood marker of pain (Achenbach et al., 2022).

Carcinoembryonic antigen-related cell adhesion molecule 1 (CEACAM1), part of the carcinoembryonic antigen cell adhesion molecule (CEACAM) family of glycosylated immunoglobulin (Ig) molecules (Kammerer and Zimmermann, 2010), is expressed across various cell types, including immune, epithelial and endothelial cells (Kammerer et al., 2017). As age advances, vascular expression of CEACAM1 escalates, and its interaction with TNF-α significantly influences the principal characteristics of aging vessels (Kleefeldt et al., 2019). It has been demonstrated in a prior study that the expression of CEACAM1 is notably elevated in Schwann cells of the sciatic nerve in rats with CCI (Ma et al., 2021), indicating that CEACAM1 may be involved in the development of NP. Analysis of immune infiltration in this research revealed a significant increase in neutrophils in the peripheral blood of NP patients compared to healthy individuals, with CEACAM1 expression showing a positive correlation with neutrophil levels. CEACAM1 serves as a indicator of neutrophil activation, contributing to the prolongation of neutrophil apoptosis (Pan and Shively, 2010) and the promotion of neutrophil adhesion to endothelial cells (Skubitz and Skubitz, 2008; Sule et al., 2020). Besides, CEACAM1 overexpression can increase the mRNA expression of CXCL6 and IL-8, thereby facilitating the recruitment of neutrophils (Wang et al., 2014). In patients with inflammatory pain, CEACAM1 on neutrophils in peripheral blood was significantly increased compared with normal people (Matsumoto et al., 2022). Prior studies have also shown that CEACAM1 on neutrophils in peripheral blood may serve as a marker for disease clinical stage (Zhang et al., 2022) and progression (Rayes et al., 2020). Further work is needed to verify whether CEACAM1 in peripheral blood can serve as a biomarker for NP.

The gene B cell transposition gene 3 (BTG3) is recognized as a tumor suppressor, known to inhibit cell cycle progression and cell proliferation (Zheng et al., 2022). The decrease in BTG3 levels can lead to upregulation of p16^INK4a^ expression via AP1-mediated transcriptional activation of JMJD3/KDM6B, ultimately inducing acute cellular senescence (Lin et al., 2012). Hypoxia promotes the progression of cellular senescence. Chronic intermittent hypoxia increases BTG3-related proteins, leading to p53 phosphorylation and nuclear retention, causing vascular endothelial cell senescence (Li et al., 2024). Following peripheral nerve injury, endoneurial hypoxia results in diminished levels of the Na/K-ATPase ion transporter, which in turn increases neural excitability and sustains mechanical hypersensitivity associated with NP (Lim et al., 2015). At present, there is no study targeting BTG3 in NP. Nevertheless, it is worth noting that hypoxia can modulate the expression of BTG3 and potentially contribute to the development of nerve damage. Under hypoxic conditions, overexpression of BTG3 inhibits the mTOR pathway and activates AMPK in neuronal cells (He et al., 2018). Additionally, targeting miR-210 suppresses BTG3 expression and activates the PI3K/AKT/mTOR pathway, thereby protecting neural stem cells from hypoxic injury (Yan et al., 2018). This indicates that BTG3 may be a valuable therapeutic target for alleviating nerve damage in NP.

The human interleukin-1 type I receptor (IL-1R1) is the signal transducing receptor for IL-1. Both the soluble and membrane-bound isoforms of IL-1R1 exhibit biological activity by modulating the inflammatory response through agonistic and antagonistic regulation of cytokine activity (Fields et al., 2019). Cell senescence associates with chronic low-grade inflammation. Previous study revealed that the interaction between IL-1 and IL-1R1 triggers the activation of NFκB, resulting in the aberrant accumulation of reactive oxygen species and mitochondrial dysfunction in oocytes, thereby expediting the process of oocyte aging (Wen et al., 2023). IL-1R1 mediates the development of NP. IL-1β engages with IL-1R1 in astrocyte, triggers the JNK/CCL2 pathway via TRAF6, and mitigates SNL-induced mechanical allodynia (Lu et al., 2014; Wang et al., 2017). Besides, intrathecal injection of IL-1R1 antagonist (IL-ra) inhibits astrocyte activation and relieves mechanical allodynia in rats with NP (Choi et al., 2019). In the present study, immune infiltration analysis showed that IL-1R1 expression was positively correlated with neutrophils. It has been found that the IL-1R1 signaling pathway promotes the recruitment of neutrophils to injured tissues through pro-survival effects on neutrophils and neutrophil-recruiting chemokines (Privratsky et al., 2018). In addition, knocking out IL-1R1 promotes neutrophil depletion in the distal stump of the sciatic nerve and significantly alleviates mechanical allodynia in rats with NP (Nadeau et al., 2011), indicating IL-1R1 expressed on neutrophils may be involved in the regulation of NP.

This study is constrained by several limitations. Firstly, the cohort size utilizing the public database is limited. Secondly, to validate the expression discrepancies and diagnostic utility of the investigated biomarkers, further *in vivo* and *in vitro* experiments are necessary. Lastly, further investigation into the association between NP mediated by CEACAM1 and IL-1R1 and neutrophil infiltration is warranted.

## 5 Conclusion

This study identified 24 DE-ARGs associated with NP in the peripheral blood, established four diagnostic biomarkers using three machine learning algorithms, and observed a positive correlation between CEACAM1 and IL-1R1 levels with neutrophils through immune infiltration analysis. This study delves deeper into the molecular mechanisms of NP, offering a foundation and direction for further research and development of pharmaceutical interventions for NP.

## Data Availability

The original contributions presented in the study are included in the article/Supplementary Material, further inquiries can be directed to the corresponding author.
